# Pharmacological mechanisms of quercetin in liver and intestinal diseases: a gut-liver axis perspective

**DOI:** 10.3389/fphar.2026.1958426

**Published:** 2026-10-01

**Authors:** Xiaozhong He, Tianyi Zhang, Yanqi Li, Yunshun Qiu, Chun Wang, Hui Wang, Liyuan Liu, Yihuan Wang, Guimin Zhang, Zhong Feng

**Affiliations:** 1 Graduate School, Tianjin University of Traditional Chinese Medicine, Tianjin, China; 2 School of Pharmacy, Shandong University of Traditional Chinese Medicine, Jinan, China; 3 Lunan Pharmaceutical Group Co., Ltd., Linyi, China

**Keywords:** bile acid, gut microbiota, gut-liver axis, pharmacological effects, quercetin

## Abstract

Hepatic and gastrointestinal disorders share interconnected pathogenic mechanisms, yet their clinical management remains challenging. This has prompted interest in therapeutic approaches targeting the gut-liver axis. Quercetin, a natural flavonoid, has been studied in this context. To assess its pharmacological effects, we searched PubMed, Web of Science, EMBASE, and the Cochrane Library for literature published since 2000 on quercetin in hepatic and intestinal diseases. This review focuses on the gut-liver axis as a framework for understanding the pharmacological effects of quercetin. Mechanistically, quercetin modulates gut-liver communication by reshaping the gut microbiota, promoting short-chain fatty acid production, and modulating bile acid metabolism. In preclinical models, it has been reported to modulate FXR/TGR5 signaling and preserve intestinal barrier integrity, thereby reducing endotoxin translocation and systemic inflammation. These effects are associated with downstream modulation of oxidative, inflammatory, and apoptotic pathways, including NF-κB, PI3K/Akt, and Nrf2. Furthermore, preclinical evidence indicates that quercetin attenuates liver injury, MASLD/MASH, fibrosis, and hepatocellular carcinoma, and exerts protective effects in models of ulcerative colitis and colorectal cancer. However, most of this evidence comes from *in vitro* and animal studies, and clinical evidence remains limited. By focusing on gut-liver crosstalk, this review provides a framework for understanding the pharmacological effects of quercetin and identifies key gaps that need to be addressed before clinical translation can be considered.

## Graphical Abstract

**Figure:**
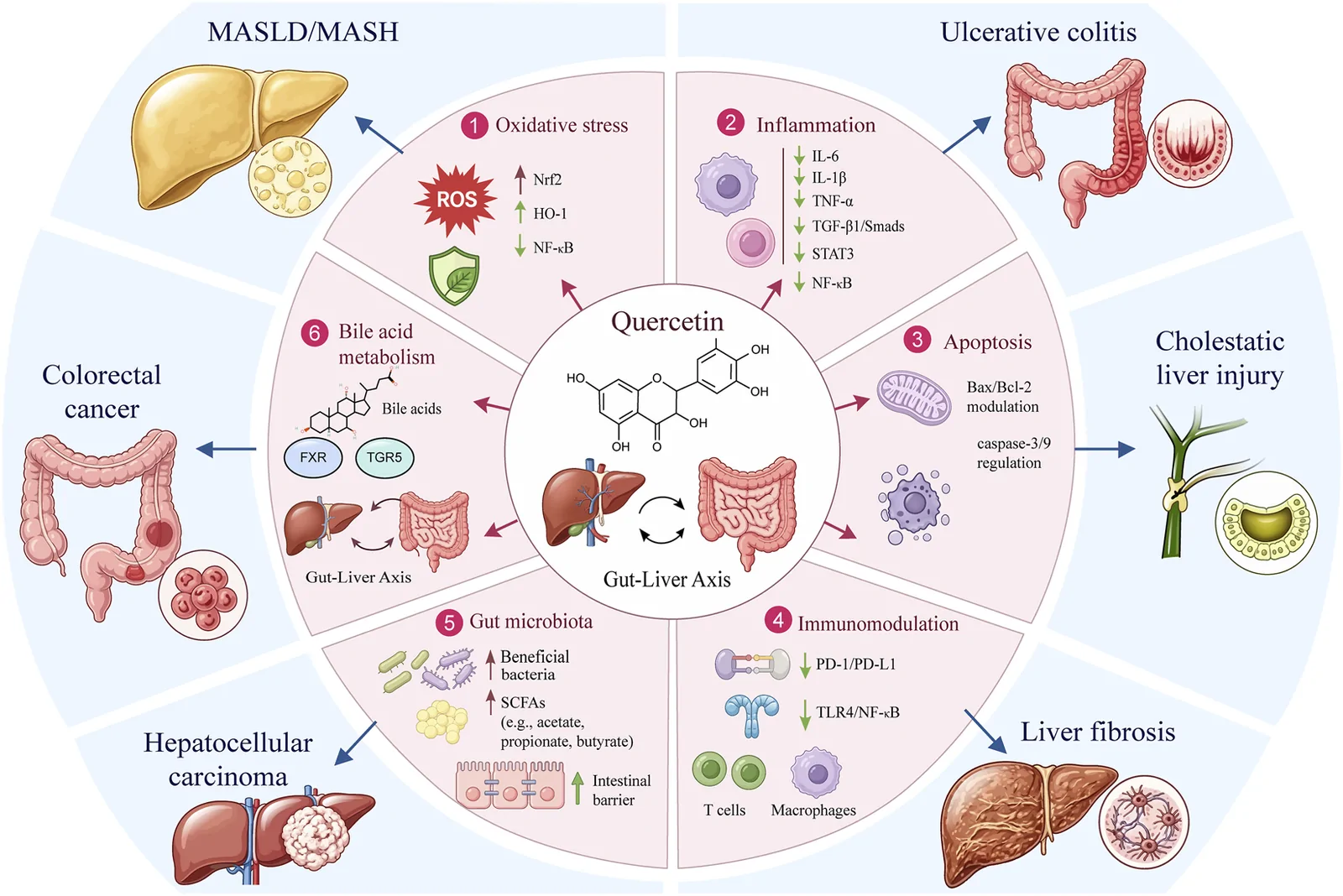

## 1 Introduction

Natural metabolites serve as valuable sources for novel drug discovery owing to their unique chemical structures and pharmacological properties. The medicinal application of these natural substances, particularly plant-derived metabolites, has historical roots in traditional Chinese medicine (TCM) (Guo and Zhang, 2025). With advances in chemistry and pharmacology, their application has become systematic, providing continuous inspiration for drug development (Liang et al., 2024; Shen et al., 2017). Natural products are studied for their pharmacological effects on various diseases. For instance, berberine is one such metabolite. It regulates blood glucose and lipid metabolism while enhancing insulin sensitivity, making it a focal point in metabolic syndrome research (Habtemariam, 2020). Curcumin has received immense attention in hepatic diseases and oncology due to its potent anti-inflammatory and antioxidant properties in pivotal organs (Farzaei et al., 2018). Ultimately, these multi-target characteristics highlight natural products as invaluable resources for novel drug discovery and advanced pharmacological research.

Quercetin (QE) is an abundant natural metabolite found in various edible and medicinal plants, such as Morus alba L. and Ginkgo biloba L. Its chemical structure features multiple phenolic hydroxyl groups, which confer biological and chemical activities (Hosseini et al., 2021). TCM has long utilized these plants to alleviate inflammation and maintain cardiovascular and hepatic health (Rather et al., 2025; Yuan and Zhao, 2017). QE is a yellow powder that is poorly soluble in water, but it is readily soluble in ethanol, methanol, and alkaline solutions (Alizadeh and Ebrahimzadeh, 2022). Even though QE is poorly absorbed by the gut, it acts on many targets and pathways to produce a wide range of pharmacological effects. These include protecting the liver, fighting oxidation, inflammation, fibrosis, and tumors. It also regulates lipid metabolism and improves insulin resistance (Mirza et al., 2023). Sul et al. showed that QE acts as an antioxidant by regulating the nicotinamide adenine dinucleotide phosphate oxidase 2 (NOX2)/reactive oxygen species (ROS)/nuclear factor kappa B (NF-κB) signaling pathway. This increases the expression of natural antioxidant enzymes and prevents the mitochondria from producing ROS (Sul and Ra, 2021). Meng et al. also reported that QE mitigates inflammation by blocking the NF-κB and mitogen-activated protein kinase (MAPK) cascades, thereby reducing the production of pro-inflammatory cytokines, such as tumor necrosis factor alpha (TNF-α), interleukin 6 (IL-6), and interleukin-1 beta (IL-1β) (Meng et al., 2018). In addition, QE attenuates fibrosis by downregulating transforming growth factor-beta (TGF-β), alpha-smooth muscle actin (α-SMA), and collagen levels, subsequently decreasing extracellular matrix (ECM) deposition (Guo et al., 2022). Its antitumor pharmacological effect is linked to the downregulation of proliferation and metastasis-related factors, consequently suppressing tumor growth and progression (Liu et al., 2021). QE has been reported to show protective effects in preclinical models of viral hepatitis (Mandal and Hazra, 2023).

The liver and intestine are pivotal organs for maintaining metabolic homeostasis and systemic immune defense. While the liver primarily regulates metabolism and clears toxins, the intestine absorbs nutrients and acts as a dynamic environmental barrier (Chelakkot et al., 2018; Yan et al., 2025). This functional cross-talk extends far beyond direct anatomical links like the enterohepatic circulation (Albillos et al., 2020). Gut-derived microbial products, such as lipopolysaccharide (LPS) and active metabolites, reach the liver via the portal vein, modulating hepatic metabolism, inflammation, and immune homeostasis (Mooser et al., 2025). Liver-derived bile acids actively shape gut microbiota composition, maintain barrier integrity, and mediate immune signaling via the farnesoid X receptor (FXR) and TGR5 pathways (Jia et al., 2018). Together with cytokines, these reciprocal interactions constitute a tightly regulated gut-liver axis (Chen et al., 2024). Clinical management of gastrointestinal and hepatic disorders remains challenging due to their complex, interacting pathologies, highlighting the need for novel therapeutics. QE has been studied as a candidate for gut-liver axis research because of its pleiotropy, its reported hepatoprotective effects in preclinical models, and its distinct pharmacokinetic profile compared to other common flavonoids. Following ingestion, QE undergoes extensive enterohepatic circulation, leading to accumulation of its phase II metabolites in the intestinal tract (Chalet et al., 2018). Within this local microenvironment, these metabolites interact directly with the gut microbiota to promote SCFA production and maintain barrier integrity. Subsequently, the biotransformed active metabolites are transported back to the liver via the portal vein (Chen et al., 2005). This distinct pharmacokinetic trajectory positions QE as a natural pharmacological bridge, seamlessly aligned with the anatomical and functional pathways of the gut-liver axis.

Several existing reviews have summarized the pharmacological effects of QE from specific perspectives, yet their overall scope remains fragmented. For instance, previous literature on liver diseases primarily highlights cell death mechanisms (Xiong et al., 2024), while reviews on inflammatory bowel disease concentrate on local intestinal effects (Lyu et al., 2022). Furthermore, although Porras et al. demonstrated the protective effects of QE on MASLD via the gut-liver axis, this framework was not extended to a broader spectrum of gastrointestinal and hepatic disorders (Porras et al., 2019; Porras et al., 2017). Rather than centering on a single disease or mechanism, the present review establishes the gut-liver axis as the core hub for the systemic pharmacological effects of QE. It synthesizes pharmacological evidence across both hepatic and intestinal disorders. Such a comprehensive cross-organ integration framework remains largely unexplored in previous reviews.

This narrative review employed a literature search strategy to identify relevant studies. To ensure comprehensive coverage, we queried four major databases: PubMed, Web of Science, EMBASE, and the Cochrane Library. Publications spanning from 1 January 2000, to June 2026 were retrieved using a combination of Medical Subject Headings and free-text terms. The Boolean search strings were constructed as follows: (“QE” OR “Quercetin”) AND (“liver disease” OR “hepatic” OR “intestinal” OR “gut” OR “hepatocellular carcinoma” OR “HCC” OR “Cholestasis” OR “liver fibrosis” OR “MASLD” OR “MASH” OR “NAFLD” OR “NASH” OR “HAV” OR “HBV” OR “Ulcerative colitis” OR “Colorectal cancer” OR “gut microbiota” OR “gut-liver axis”) AND (“mechanism” OR “signaling pathway” OR “target” OR “synergistic” OR “sensitizer”). In alignment with the updated nomenclature from the 2023 multi-society consensus, this manuscript adopts the terms metabolic dysfunction-associated steatotic liver disease (MASLD) and steatohepatitis (MASH). Previous designations, specifically NAFLD and NASH, are retained exclusively in direct quotations or historical citations (Rinella et al., 2023).

To ensure comprehensive coverage, the following inclusion criteria were applied: (1) peer-reviewed English publications; (2) original research articles (*in vivo* and *in vitro* models) and relevant reviews; and (3) studies primarily elucidating the specific pharmacological effects and molecular targets of QE. Although monotherapy remained our primary focus, we also included studies exploring the synergistic or chemosensitizing effects of QE combined with specific agents (such as sorafenib or cisplatin). This inclusion specifically highlights its potential as an adjuvant therapy. The exclusion criteria comprised: (1) studies employing complex, uncharacterized botanical decoctions where the distinct contribution of QE remained unclear; and (2) conference abstracts, short communications, or non-peer-reviewed preprints lacking complete experimental data.

## 2 Hepatoprotective effects against liver diseases

Table 1 comprehensively summarizes the experimental models, effective dosages, and targeted signaling pathways underlying the hepatoprotective pharmacological effects of QE.

**TABLE 1:** Information on the protective effects of QE on the liver.

Disease	Animal/cell model	Dose	Targets/pathways/mechanisms	References
Liver injury	Acrylamide-induced injury	50 mg/kg	ROS, MDA, and GSH	(Huang et al., 2023)
	Acrylamide-induced injury	25 mg/kg	IL-1β, IL-6, Bax, caspase-3; MAPK/NF-κB/NLRP3 pathways	(Atta et al., 2025)
	APAP-induced injury	50 mg/kg	Nrf2/ARE pathway	(Truong et al., 2016)
	L02 normal human liver cells	50 μmol/L	Nrf2 pathway	(Ji et al., 2015)
	Lead-induced injury	10 mg/kg	Bax, caspase-3, Bcl-2; JNK pathway	(Liu et al., 2010)
	L02 normal human liver cells	10 μmol/L	Bax, p53, caspase-3, Bcl-2; SIRT1/NLRP3 pathways	(Zhang et al., 2020)
	HepG2 cells	400 μmol/L	IL-6	(Lin et al., 2024)
	AML12 hepatocytes	50 μmol/L	ROS, MMP	(Wu et al., 2024)
	LPS/iE-DAP-induced injury	50 mg/kg	IL-6/STAT3 pathway	(Zhang et al., 2025)
Cholestasis	BDL-induced cholestasis	30 mg/kg	Rac1, Rac1-GTP, NOX1, MDA; Rac1/NOX1 pathway	(Kabirifar et al., 2017)
	BDL-induced cholestasis	25 mg/kg	MyD88, TAK1, MDA, IL-1β, MCP-1; NF-κB and TGF-β/Smad2/3 pathways	(Lin et al., 2014)
	ANIT-induced cholestasis	50 mg/kg	Nrf2, HO-1, PPAR-γ; TLR4/NF-κB and NLRP3/caspase-1/IL-1β pathways	(Fawzy et al., 2023)
Liver fibrosis	CCl_4_-induced fibrosis	50 mg/kg	Col3a1, Col4a1, CTGF, TNF-α, IL-1β, and IL-6	(Li et al., 2018)
	CCl_4_-induced fibrosis	15 mg/kg	Bcl-2, Bax, caspase-3, MMP-1, TIMP-1; NF-κB and p38 MAPK pathways	(Wang et al., 2017)
	BDL- and CCl_4_-induced mouse models	100 and 200 mg/kg	TGF-β1/Smads and PI3K/Akt pathways; autophagy	(Wu et al., 2017b)
	Hepatic stellate cells	20–40 μmol/L	Bcl-2, Bax/Bcl-2 ratio, cytochrome c, caspase-9, and caspase-3	(He et al., 2016)
	EAC cell-induced fibrosis	50 mg/kg	ALT and AST	(Sannappa Gowda et al., 2023)
	HFCD-induced MASLD-related fibrosis	50 mg/kg	IL-1β, iNOS, CD45, p53, TIMP-1, and α-SMA	(Alqahtani et al., 2026)
MASLD/MASH	HFD-induced MASLD	50 mg/kg	IL-6 and TNF-α	(Katsaros et al., 2024)
	MCD-induced MASH	20–80 mg/kg	PINK1 and Parkin	(Cao et al., 2023)
	MCD-induced MASH	50 mg/kg	IL-6, TNF-α, and TLR4	(Marcolin et al., 2012)
	HFD-induced MASLD	50–100 mg/kg	Nrf2, HO-1, PGC-1α, Nrf1, Tfam; Nrf2/HO-1 pathway	(Kim et al., 2015)
	High glucose and free fatty acid-induced MASLD	5 μmol/L (*in vitro*)	SREBP-1, XBP-1, p-AMPK, and p-ACACA	(Gnoni et al., 2022)
	MCD diet-induced MASLD	50 mg/kg	TNF-α, SOCS3, iNOS, FABP1, FATP5, and PPARα	(Pisonero-Vaquero et al., 2015)
HCC	H22 tumor-bearing BALB/c mice	25–100 mg/kg	NF-κB pathway	(Wu et al., 2022)
	LM3 cells	120 μmol/L	PCNA, Cyclin B1, Bax; JAK2/STAT3 pathway	(Wu et al., 2019)
	SMMC7721 and HepG2 cells	80 μmol/L	p62, Bcl-2, LC3-II, Beclin-1, Bax, and caspase-3	(Ji et al., 2019)
	MRHM-induced early-stage HCC model	10 mg/kg	α-SMA, OV6, and EpCAM	(Reyes-Avendaño et al., 2022)
	HCC tumor-bearing model	100 mg/kg	PD-1/PD-L1 pathway	(Lu et al., 2025)
Viral hepatitis	LMH cells	40 μmol/L	METTL3, PTEN, Bax, cytochrome c; PI3K/Akt pathway	(Xia et al., 2025)
	HuH7 and HepG2.2.15 cells	50 μmol/L	HBsAg, HBeAg, and heat shock proteins	(Cheng et al., 2015)
	HepG2.2.15 cells	150 μmol/L	HBsAg, HBeAg, and CDK1	(Hao et al., 2024)
	BALB/c mice	50 mg/kg	PI3K/Akt/GSK3β signaling axis	(Wang et al., 2026)
	BALB/c mice	100–200 mg/kg	TNF-α, IL-6, and IFN-γ	(Wu et al., 2017a)
	BALB/c mice	50 mg/kg	ALT and AST	(Domitrović et al., 2012)

### 2.1 Alleviation of liver injury

The liver serves as a central metabolic and detoxifying organ. It plays key roles in nutrient metabolism, bile acid synthesis, xenobiotic biotransformation, and immune homeostasis (Rui, 2014). Owing to its distinct anatomical position and physiological functions, the liver is continuously exposed to endogenous metabolites and exogenous toxins, rendering it highly susceptible to injury (Wang et al., 2025). Liver injury is often caused by factors, such as viral infections, alcohol abuse, drug-induced injury, a high-fat diet (HFD), metabolic disorders, and environmental toxins. These factors lead to liver cell damage, stress, inflammation, and metabolic dysregulation (Devarbhavi et al., 2023). If liver damage is not treated, it will progress to chronic liver disease, posing a serious clinical challenge (Hyun et al., 2021).

In an acrylamide-induced liver injury model, 50 mg/kg QE protected the liver by decreasing oxidative stress. This was shown by reduced levels of ROS and malondialdehyde (MDA), and restored glutathione (GSH) levels. Concurrently, QE inhibited ferroptosis by binding to nuclear receptor coactivator 4 (NCOA4). This interaction prevents ferritin heavy chain 1 (FTH1) degradation and subsequently reduces intracellular free iron. However, the binding of QE to NCOA4 was evidenced mainly by molecular docking, with no biophysical validation such as cellular thermal shift assay or surface plasmon resonance to confirm the physical interaction. Whether NCOA4 is a direct target of QE still requires further verification (Huang et al., 2023). In a similar model, Atta et al. showed that 25 mg/kg QE nanoparticles demonstrated hepatoprotective effects by suppressing the MAPK, NF-κB, and NOD-like receptor family pyrin domain containing 3 (NLRP3) inflammatory pathways. This suppression downregulated inflammatory markers such as IL-1β and IL-6, as well as apoptotic proteins like Bax, cysteine-aspartic acid protease 3 (caspase-3), and p53, while bolstering endogenous antioxidant defenses. A methodological limitation in this study is the dose discrepancy between the nanoformulation and the free compound. Whether the pharmacological effect observed reflects an advantage of the nanoformulation itself, rather than simply the higher dose given, thus remains uncertain (Atta et al., 2025). The pharmacological effects of QE have also been studied in models of APAP-induced hepatotoxicity, a process involving the activation of the nuclear factor erythroid 2-related factor 2 (Nrf2) pathway. Ji et al. found that 25–50 μmol/L QE promoted Nrf2 nuclear translocation and increased Nrf2-mediated ARE activity in APAP-treated normal human liver L-02 cells. APAP alone weakly increased ARE activity, and QE further elevated this activity. However, this evidence is derived from an *in vitro* L-02 cell model, with APAP used at 7.5 mM. Whether the same mechanism operates *in vivo* remains to be further investigated (Ji et al., 2015). Cytochrome P450 2E1 (CYP2E1) is a key enzyme in the liver, because it can metabolize drugs (Truong et al., 2016). In a model of liver damage caused by APAP, 20 mg/kg QE reduced liver injury by inhibiting CYP2E1 activity and reducing the production of the toxic metabolite N-acetyl-p-benzoquinone imine (NAPQI). It should be noted that this conclusion is based solely on data from rats and isolated rat hepatocytes. Since CYP enzyme profiles, particularly CYP2E1 expression and substrate specificity, are known to differ between rodents and humans, direct extrapolation of these findings to the clinical setting may be limited. Therefore, while the proposed CYP2E1-mediated protective mechanism is plausible, its relevance to patients still requires further verification (Pingili et al., 2019).

In addition to the APAP- and acrylamide-induced models, QE has also been studied in other experimental models of liver injury. In a study of lead-induced liver injury in rats, 10 mg/kg QE alleviated hepatic damage by downregulating the expression of pro-apoptotic proteins such as Bax, caspase-3, and phosphorylated c-Jun N-terminal kinase 1/2 (p-JNK1/2), while upregulating the anti-apoptotic protein B-cell lymphoma 2 (Bcl-2). Importantly, these observations are primarily correlative; the precise causal role of the JNK pathway remains to be established using specific pharmacological inhibitors or genetic manipulation (Liu et al., 2010). *In vitro* studies further corroborated the anti-apoptotic pharmacological effects of QE against hepatic damage. For instance, Zhang et al. demonstrated that 10 μmol/L QE protected L02 human hepatocytes from damage caused by isoniazid. This protection was mediated by the sirtuin 1 (SIRT1)/NLRP3 pathway, which upregulated Bcl-2 and downregulated Bax, p53, and caspase-3 expression. The involvement of SIRT1 was established primarily through the inhibitor EX527, and whether direct SIRT1 activation alone can reproduce these protective effects remains to be further clarified (Zhang et al., 2020). Despite these mechanistic gaps, the consistent suppression of hepatocyte apoptosis in both the *in vivo* rat model and the *in vitro* hepatocyte model suggests that inhibiting apoptosis may be an important mechanism underlying QE-mediated alleviation of liver injury.

Lin et al. demonstrated that 400 μmol/L QE reversed high glucose-induced epithelial-mesenchymal transition (EMT) in HepG2 cells. Because EMT is closely linked to liver fibrosis and inflammation, this effect was achieved through reduced IL-6 expression, highlighting the potential of QE to reduce liver damage. This finding’s translational relevance is constrained by the application of 400 μmol/L QE, a supra-physiological concentration that vastly exceeds achievable plasma levels *in vivo* (Lin et al., 2024). In a model of LPS/D-galactosamine-induced AML12 hepatocyte injury, 50 μmol/L QE reduced apoptosis by restoring mitochondrial membrane potential, suppressing ROS accumulation and facilitating cellular repair. However, this study was limited to the AML12 cell line, and whether primary hepatocytes respond to QE in a similar manner has not yet been examined (Wu et al., 2024). QE-preconditioned BMSC-derived EVs delivered miR-136-5p, which targeted and inhibited the GNAS/phosphoinositide 3-kinase (PI3K)/extracellular signal-regulated kinase (ERK)/signal transducer and activator of transcription 3 (STAT3) pathways. This inhibition changed macrophage polarization from the M1 type to the M2 type, reducing hepatic inflammation and protecting the liver. Furthermore, the functional validation of miR-136-5p and GNAS was restricted to the RAW264.7 cell line, leaving their essentiality *in vivo* unverified (Jiang X. et al., 2024). *In vivo*, 50 mg/kg QE reduced LPS/iE-DAP-induced acute liver injury by targeting the IL-6/STAT3 signaling pathway. This effect was accompanied by the inhibition of ferritinophagy and reductions in hepatic iron accumulation and lipid peroxidation. As a result, QE preserved hepatic architecture and function. A notable caveat of this mechanistic model is that the link between STAT3 activation and ferritinophagy was inferential. Direct evidence demonstrating STAT3-mediated transcriptional regulation of ferritinophagy-related genes is currently lacking (Zhang et al., 2025).

### 2.2 Cholestasis regulation

Cholestasis is a pathological condition. This condition is characterized by impaired bile formation, secretion, or excretion. As a result, bile acids and other potentially toxic metabolites accumulate abnormally in the liver (Elferink, 2003). Based on the anatomical site of the lesion or obstruction, the condition is classified into two forms: intrahepatic and extrahepatic. Intrahepatic cholestasis originates from various factors such as hepatocellular dysfunction, defective canalicular transport, or genetic and metabolic disorders, while the latter originates from mechanical biliary tract obstruction, including gallstones, tumors, or strictures (Clements et al., 2020). A range of causes can trigger this condition. These include genetic susceptibility, drug-induced liver injury, pregnancy, autoimmune liver diseases, and biliary obstruction. The condition disrupts bile acid balance and impairs bile flow (Smith and Rood, 2020). Without effective treatment, persistent cholestasis can result in progressive liver dysfunction and severe liver damage (Tacke, 2017).

QE exerts hepatoprotective effects against cholestasis through multiple mechanistic pathways. Oxidative stress drives cholestasis progression, with the Ras-related C3 botulinum toxin substrate 1 (Rac1)/NADPH oxidase 1 (NOX1) pathway acting as a primary signaling axis for ROS generation. Kabirifar et al. identified the modulation of the Rac1, Rac1-GTP, and NOX1 axis as a core mechanism underlying the pharmacological effects of QE against cholestatic liver injury. In a bile duct ligation (BDL)-induced cholestasis rat model, administration of 30 mg/kg QE downregulated Rac1, Rac1-GTP, and NOX1 expression at both the mRNA and protein levels. This intervention enhanced the activities of antioxidant enzymes such as superoxide dismutase (SOD) and catalase (CAT), decreased MDA and protein carbonylation, and restored thiol content, ultimately attenuating BDL-induced hepatic fibrosis. However, these mechanistic conclusions were derived from expression changes in Rac1, Rac1-GTP, and NOX1. Further studies using specific inhibitors or gene knockdown approaches would help establish whether suppression of this axis is required for the hepatoprotective effects of QE (Kabirifar et al., 2017). Additional research demonstrated that 25 mg/kg QE mitigated BDL-induced cholestatic liver injury by suppressing myeloid differentiation factor 88 (MyD88) and transforming growth factor-β-activated kinase 1 (TAK1)-mediated NF-κB activation. This intervention also blocked the pro-fibrotic TGF-β/Smad2/3 signaling pathway, reduced MDA levels, elevated GSH content and CAT activity, and suppressed the production of pro-inflammatory cytokines such as IL-1β and monocyte chemoattractant protein-1 (MCP-1). It should be noted that these findings were derived from changes in protein expression and pathway activity, and whether these pathways are required for the hepatoprotective effects of QE remains to be further established (Lin et al., 2014). Fawzy et al. found that QE exhibited synergistic hepatoprotective effects when combined with sildenafil or pentoxifylline. This combination therapy improved α-naphthylisothiocyanate (ANIT)-induced acute intrahepatic cholestasis. This effect was achieved by upregulating Nrf2, heme oxygenase-1 (HO-1), and peroxisome proliferator-activated receptor gamma (PPAR-γ) expression, while concurrently inhibiting the Toll-like receptor 4 (TLR4)/NF-κB inflammatory cascade and the NLRP3/caspase-1/IL-1β pyroptosis pathway. Co-administration of 50 mg/kg QE with either 10 mg/kg sildenafil or 50 mg/kg pentoxifylline demonstrated greater pharmacological effects over monotherapy in reducing serum transaminase and bilirubin levels and alleviating histopathological liver damage. These findings suggest that QE may have multifaceted pharmacological effects in cholestasis. This study was limited to the ANIT-induced cholestasis model, and whether the same mechanisms operate in other forms of cholestasis remains unclear (Fawzy et al., 2023).

### 2.3 Alleviation of liver fibrosis

Liver fibrosis is a pathological condition defined by the excessive deposition of ECM, typically arising from chronic hepatic injury and disordered wound-healing responses (Bataller and Brenner, 2005). Common causes include viral infections, alcohol abuse, metabolic disorders, drug toxicity, and cholestatic diseases, all of which can lead to persistent hepatic damage (Zhao et al., 2022). The pathogenesis of this fibrotic state is closely associated with the activation of hepatic stellate cells, which are primary cellular drivers of liver disease (Higashi et al., 2017). If unmanaged, progressive liver fibrosis provides the base for the transition of chronic liver diseases into cirrhosis and hepatocellular carcinoma (Liu and Yang, 2023).

A previous study indicated that 50 mg/kg QE downregulates the expression of fibrotic genes such as Col3a1, Col4a1, and Ctgf, as well as pro-inflammatory cytokines including TNF-α, IL-1β, and IL-6. This intervention enhances hepatic anti-inflammatory and antioxidant capacities, reducing CCl_4_-induced liver fibrosis in mice. The same study revealed that QE inhibited hepatic stellate cell activation *in vitro*, downregulated Notch1 expression, and inhibited inflammatory cytokine release. It should be noted that the link between Notch1 downregulation and the anti-fibrotic effects of QE was inferred from parallel changes in Notch1 expression and fibrotic markers. Further work is needed to determine whether Notch1 is required for these protective effects in this study (Li et al., 2018). Utilizing a comparable CCl_4_-induced model, Wang et al. found that 15 mg/kg QE had anti-fibrotic effects by inhibiting the NF-κB and p38 MAPK inflammatory pathways and regulating the Bcl-2/Bax/caspase-3 apoptotic cascade. This QE administration alleviated liver injury and collagen accumulation as well as promoted ECM degradation through the restoration of the balance between matrix metalloproteinase 1 (MMP-1) and tissue inhibitor of metalloproteinases 1 (TIMP-1). However, QE was administered by intraperitoneal injection in this study, a route that bypasses intestinal absorption and first-pass metabolism and therefore does not reflect the oral exposure typical of dietary or supplemental QE intake in humans (Wang et al., 2017). In addition to the CCl_4_ model, QE has also been studied in BDL-induced liver fibrosis. *In vivo* studies showed that QE (100 and 200 mg/kg) attenuated liver fibrosis in both BDL- and CCl_4_-induced mouse models by reducing hepatic TGF-β1, p-Smad2, and p-Smad3 expression, while increasing PI3K and p-Akt levels, thereby inhibiting hepatic stellate cell activation and autophagy. A limitation of this study is that the effects of QE on hepatic stellate cell activation and autophagy were inferred from liver tissue analysis rather than from isolated hepatic stellate cell experiments (Wu et al., 2017b). Subsequent cellular studies found that 20–40 μmol/L QE not only induced mitochondrial apoptosis in hepatic stellate cells but also attenuated liver fibrosis. This process involved decreasing Bcl-2 expression, elevating the Bax/Bcl-2 ratio and cytochrome c release, and activating caspase-9 and caspase-3. QE activated the endoplasmic reticulum (ER) stress pathway by promoting protein kinase R-like ER kinase (PERK) and inositol-requiring enzyme 1 (IRE1) phosphorylation, and promoting activating transcription factor 6 (ATF6) cleavage. These findings were obtained *in vitro*, and whether QE induces apoptosis and attenuates liver fibrosis through this ER stress-mediated mechanism has not been examined *in vivo* (He et al., 2016).

In another model, administration of 50 mg/kg QE alleviated Ehrlich ascites carcinoma cell-induced liver fibrosis. This protective effect was mediated by inhibiting tumor angiogenesis and enhancing hepatic antioxidant defenses. This intervention reduced serum ALT and AST activities, inflammatory cell infiltration and hepatic collagen deposition. However, the antioxidant defense mechanisms were inferred from reduced liver enzyme levels and improved histopathology, as direct measurements of hepatic oxidative stress markers were not included in this study (Sannappa Gowda et al., 2023). Al-Ani et al. demonstrated that 50 mg/kg QE mitigated high-fat and high-cholesterol diet (HFCD)-induced MASLD-related liver fibrosis. QE inhibited the IL-1β/inducible nitric oxide synthase (iNOS) and IL-1β/CD45 inflammatory axes, downregulated p53-mediated apoptotic signaling alongside fibrotic markers including TIMP-1 and α-SMA, and restored hepatic antioxidant capacity. The absence of a QE-only control group in this experimental design compromises the ability to differentiate the intrinsic pharmacological properties of QE on hepatic fibrotic signaling from its protective actions against HFCD-induced fibrosis (Alqahtani et al., 2026).

### 2.4 Amelioration of metabolic dysfunction-associated steatotic liver disease/steatohepatitis

Metabolic dysfunction-associated steatotic liver disease (MASLD) is a recently approved nomenclature designed to broaden diagnostic criteria and avoid the stigmatization associated with nonalcoholic fatty liver disease (NAFLD) (Rinella et al., 2023). This term encompasses a spectrum of conditions ranging from simple hepatic steatosis to metabolic dysfunction-associated steatohepatitis (MASH). The latter involves inflammation and carries a high risk of progressive liver damage (Sanyal, 2002). The global MASLD prevalence is approximately 25%. Its incidence continues to rise rapidly (Lebeaupin et al., 2018). The disease encompasses a pathophysiological spectrum ranging from simple steatosis to MASH, constituting a major global health burden (Velliou et al., 2023).

QE is a natural product with antioxidant and anti-inflammatory properties. A number of preclinical studies have reported pharmacological effects of QE in animal models of MASLD and MASH. In models of HFD-induced MASLD, Katsaros and colleagues found that 50 mg/kg QE mitigated hepatic steatosis and steatohepatitis by suppressing pro-inflammatory factors such as IL-6 and TNF-α (Katsaros et al., 2024). QE at 20–80 mg/kg regulated the serum acylcarnitine metabolic profile and upregulated the expression of key mitophagy-related proteins such as PTEN-induced putative kinase 1 (PINK1) and Parkin. This regulation alleviated three key pathological features induced by a methionine- and choline-deficient (MCD) diet. These included hepatic lipid accumulation, oxidative stress, and inflammatory responses (Cao et al., 2023). Another study utilized the same MCD model and found that 50 mg/kg QE inhibited hepatic lipid accumulation. This effect was achieved by suppressing the TLR4 signaling pathway and reducing IL-6 and TNF-α production, highlighting its anti-inflammatory pharmacological profile in experimental MASLD models (Marcolin et al., 2012). However, both studies relied on the MCD diet model, whose metabolic phenotype differs from human MASLD/MASH. As acknowledged by the authors themselves, MCD-fed mice exhibit weight loss and enhanced insulin sensitivity, features that contrast with the obesity and insulin resistance seen in patients. Since this model represents an acute nutritional stress rather than the chronic metabolic syndrome background of human disease, the protective effects of QE should be extrapolated to clinical MASLD/MASH with caution. Kim et al. reported that administering QE at doses ranging from 50 mg/kg to 100 mg/kg exerted pharmacological effects against HFD-induced MASLD by upregulating the expression of Nrf2 and HO-1, as well as mitochondrial biogenesis-related genes such as PGC-1α, Nrf1, and Tfam. These molecular alterations correlate with reduced hepatic triglyceride content, decreased levels of the lipid peroxidation marker thiobarbituric acid reactive substances (TBARS), and lowered serum ALT levels. The protective effects of QE were partially abolished by the HO-1 inhibitor zinc protoporphyrin (ZnPP) and further negated in Nrf2-deficient mice, indicating that its anti-steatotic properties are primarily mediated through the Nrf2/HO-1 signaling pathway. The Nrf2-deficient mice used in this study were on a B6/SJL background, whereas the wild-type controls were on a C57BL/6 background. This genetic background difference may have influenced the observed responses to QE (Kim et al., 2015).

In a cellular model of MASLD induced by high glucose and free fatty acids, 5 μmol/L QE inhibited hepatocellular lipid accumulation by downregulating sterol regulatory element-binding protein 1 (SREBP-1) and X-box binding protein 1 (XBP-1) expression. This regulation concurrently upregulated p-AMPK and phosphorylated acetyl-CoA carboxylase alpha (p-ACACA) levels, attenuating free fatty acid-induced lipotoxicity. These mechanistic findings were obtained in a cellular model. However, *in vivo* evidence is lacking to confirm that QE suppresses *de novo* lipogenesis through the SREBP-1/XBP-1 and AMPK/ACACA axis in the liver (Gnoni et al., 2022). Utilizing a MCD diet-induced MASLD mouse model, 50 mg/kg QE relieved hepatic steatosis and inflammation. This improvement was demonstrated through the downregulation of pro-inflammatory genes such as TNF-α, suppressor of cytokine signaling 3 (SOCS3), and iNOS. It upregulated lipid metabolism-related transcription factors such as fatty acid-binding protein 1 (FABP1), fatty acid transport protein 5 (FATP5), and peroxisome proliferator-activated receptor alpha (PPARα). *In vitro* studies also found that 10 μmol/L QE decreased lipid accumulation and peroxidation in Huh7 cells by inhibiting PI3K and Akt phosphorylation, reinforcing its pharmacological effects against MASLD (Pisonero-Vaquero et al., 2015).

A meta-analysis involving 7 RCTs (540 patients with MASLD) showed that QE reduced ALT, AST, GGT and CRP, and improved the lipid profile. However, most of the core indicators in this analysis had high heterogeneity, and the GRADE evidence quality was extremely low to moderate. Moreover, all studies were based on serological endpoints, lacked histological assessment, had short intervention periods, and some were subject to confounding by combined medication. This suggests that QE shows a preliminary protective signal in MASLD, and further validation is needed with large sample sizes, long intervention periods, histological endpoints, and RCTs (Jin et al., 2025). On this basis, a double-blind, randomized, placebo-controlled phase 1/2 clinical trial (NCT05506488) has been conducted to explore the population with MASLD-related fibrosis, including 30 patients, with the intervention regimen of dasatinib (100 mg) combined with QE (1,000 mg). This trial took an approach from the perspective of eliminating senescent cells (Senolytics), expanding the new direction for the clinical application of QE.

### 2.5 Hepatocellular carcinoma inhibition

Hepatocellular carcinoma (HCC) is the most common type of primary liver cancer. It accounts for approximately 80% of cases and is one of the top causes of cancer deaths globally (Yeo et al., 2025). Its incidence continues to escalate rapidly, a trend largely driven by the surging global prevalence of obesity and metabolic disorders (Lee et al., 2024). Contemporary clinical management encompasses diverse modalities, including surgical resection, liver transplantation, local ablative therapies, transarterial chemoembolization, targeted therapies, and immunotherapy (Vibert et al., 2020). The clinical efficacy of these treatments faces considerable challenges. These arise from high tumor heterogeneity, rapid recurrence and metastasis, therapeutic resistance, and considerable adverse effects (Su et al., 2016). These clinical limitations highlight the need for novel and safe therapeutic approaches. Preclinical studies have reported anti-tumor effects of QE in HCC cell and animal models.

Wu et al. reported that in H22 tumor-bearing BALB/c mice 25–100 mg/kg QE promoted the polarization of tumor-related macrophages toward an M1-like phenotype, improving autophagy in cancer cells and exerting dose-dependent anticancer effects. This antitumor activity is linked to the inhibition of the NF-κB signaling pathway. However, whether M1 polarization is causally required for the antitumor effects of QE was not tested using macrophage depletion or polarization inhibitors (Wu et al., 2022). In LM3 cells, 120 μmol/L QE induced apoptosis and autophagy. This effect was mediated by the inhibition of the JAK2/STAT3 signaling pathway. It also involved reduction in proliferating cell nuclear antigen (PCNA) and cyclin B1 expression, and the upregulation of Bax (Wu et al., 2019). Ji et al. reported similar apoptotic and autophagic responses in SMMC7721 and HepG2 cells treated with 80 μmol/L QE. This process involved the downregulation of p62 and Bcl-2, and the upregulation of microtubule-associated protein 1 light chain 3-II (LC3-II), Beclin-1, Bax, and caspase-3. Beyond cellular apoptosis induction, QE is also a key metabolite of the tumor microenvironment during the early stages of HCC (Ji et al., 2019). Administration of 10 mg/kg QE suppressed early HCC progression. This effect was achieved by diminishing preneoplastic lesion areas, decreasing collagen deposition and α-SMA-positive myofibroblasts, and downregulating cancer stem cell markers such as oval cell marker 6 (OV6) and epithelial cell adhesion molecule (EpCAM). The clinical targeting of the PD-L1/PD-1 immune checkpoint is a central strategy in modern cancer immunotherapy. However, the animal experiments were conducted with only four rats per group in this study, which limits the statistical power to detect smaller effect sizes (Reyes-Avendaño et al., 2022). Lu et al. reported that intervention with 100 mg/kg QE decreased intratumoral lactate levels and Treg infiltration in HCC. This metabolic and immunological modulation made the PD-1/PD-L1 pathway sensitive to checkpoint inhibition. This improved the effects of anti-PD-1 antibodies and enhanced T-cell-mediated antitumor immunity in HCC (Lu et al., 2025).

Combining QE with conventional therapeutic agents has attracted significant interest. Abdu et al. demonstrated that combining QE with 7.5 mg/kg sorafenib synergistically suppressed tumor growth and alleviated inflammation across HepG2 cells and HCC rat models, indicating that QE may enhance the effects of sorafenib in preclinical models (Abdu et al., 2022). The pharmacological effects of QE can be enhanced when used in combination with cisplatin. Zhao et al. demonstrated that the co-intervention of HepG2 cells with 50 μmol/L QE and 10 μmol/L cisplatin could inhibit cell proliferation and trigger apoptosis. These findings suggest enhanced antitumor pharmacological effects of this combination therapy (Zhao et al., 2014). Another study revealed that the combination of 10 μmol/L QE with 2 MOI ZD55-TRAIL in HCC cells suppressed cell proliferation and induced apoptosis. The underlying mechanism involved NF-κB pathway inactivation and modulation of apoptosis-related protein expression (Zou et al., 2018). Collectively, these studies suggest that QE may enhance the antitumor effects of conventional therapeutic agents such as sorafenib, cisplatin, and ZD55-TRAIL. The molecular basis of these synergistic effects remains incompletely understood. Whether QE potentiates these agents through direct pathway crosstalk or through pharmacokinetic interactions has not been fully elucidated.

### 2.6 Viral hepatitis prevention

Viral hepatitis is a major global infectious disease. The primary causes are hepatotropic viruses, including HAV and HBV (Wörns et al., 2008). Liver-specific proteins and immune complex-mediated hypersensitivity reactions play critical roles in the pathogenesis of this disease (Lim et al., 2020). A growing body of evidence in preclinical models suggests that QE exerts protective effects against viral hepatitis by reducing HBsAg, HBeAg, and HBV DNA levels, while modulating oxidative stress, inflammatory responses, and apoptosis in hepatocytes.

Xia et al. found that 40 μmol/L QE alleviated hepatitis. This effect targeted the METTL3/PTEN m^6^A methylation axis and activated the PI3K/Akt pathway, downregulating apoptosis-related proteins such as Bax and cytochrome c and suppressing LPS-induced apoptosis and necroptosis in LMH cells. These mechanistic findings were obtained in chicken LMH cells. Whether the METTL3/PTEN m^6^A/PI3K/Akt axis mediates comparable protective effects in human hepatocytes or human liver disease models remains to be determined (Xia et al., 2025). Additionally, Cheng et al. reported that 50 μmol/L QE inhibited HBV transcription and replication. This intervention reduced HBsAg and HBeAg secretion by downregulating heat shock proteins in HuH7 and HepG2.2.15 cells. Cyclin-dependent kinase 1 (CDK1) is a key regulator of the G2/M phase transition. It is closely associated with tumor progression and poor prognosis. Hao et al. demonstrated that 150 μmol/L QE inhibited HBsAg and HBeAg secretion in HepG2.2.15 cells by downregulating CDK1 expression. Cheng et al. and Hao et al. both reported that QE reduced HBsAg and HBeAg secretion in HepG2.2.15 cells, attributing this to heat shock protein downregulation and CDK1 inhibition, respectively. Crucially, neither study provided functional validation via genetic overexpression or knockdown approaches, rendering the causative roles of these targets inconclusive (Cheng et al., 2015; Hao et al., 2024). Another study revealed that 50 mg/kg QE suppressed tumor growth in a HepG2-HBx xenograft mouse model by targeting EGFR and reversing the EMT. *In vitro*, the PI3K/Akt/GSK3β axis was identified as the downstream pathway (Wang et al., 2026). Beyond viral inhibition, 100–200 mg/kg QE alleviated hepatocyte necrosis and apoptosis in male BALB/c mice by attenuating elevated plasma cytokines, including TNF-α, IL-6, and interferon-gamma (IFN-γ) (Wu et al., 2017a). These hepatoprotective properties were further validated in CCl_4_-intoxicated male BALB/c mice, where 50 mg/kg QE reduced serum ALT and AST levels and alleviated hepatic oxidative stress (Domitrović et al., 2012).

## 3 Protection against gut-related diseases

Table 2 comprehensively outlines the specific *in vivo* and *in vitro* models, applied dosages, and molecular mechanisms mediating the protective pharmacological effects of QE against intestinal pathologies.

**TABLE 2:** Information on the protective effects of QE on the gut.

Disease	Animal/cell model	Dose	Targets/pathways/mechanisms	References
UC	DSS-induced UC	50 mg/kg	IL-6, IL-17A, Ly6G, SOD2; NF-κB pathway	(Wei et al., 2025)
	DSS-induced UC	10 mg/kg	TNF-α, IL-1β; NF-κB pathway	(Jiang et al., 2024b)
	Ochratoxin A-induced UC	100 mg/kg	MDA, NO, MPO, Hydroxyproline, SOD, GSH, OPN, and IL-6	(Yin et al., 2023)
	DSS-induced UC	25–100 mg/kg	ZO-1, Claudin-1, and AhR	(Wang et al., 2024)
	DSS-induced UC	100 mg/kg	TNF-α, IL-6, IL-1β; PI3K/Akt pathway	(Zhang et al., 2022)
	DSS-induced UC	30 mg/kg	iNOS, TNF-α, IFN-β, CXCL10, Arg1, IL-10, CCL17; cGAS-STING pathway	(Gao et al., 2024)
CRC	HT-29 cells	50–200 μmol/L	CSN6, c-Myc, p53, Bax, Bcl-2; PI3K/Akt pathway	(Yang et al., 2016)
	HT-29 cells	40 μmol/L	COX-2, iNOS, ROS; NF-κB pathway	(Raja et al., 2017)
	Caco-2 and DLD-1 cells	50 μmol/L	β-catenin, c-Myc; PI3K/Akt/mTOR and JNK/JUN pathways	(Refolo et al., 2015)
	HCT-116 cells	150 μmol/L	LRG1; PI3K/Akt and Ras pathways	(Zhang et al., 2019)
	AOM/DSS-induced CRC	30 mg/kg	LPO, NO, SOD, G6PD, and GSH	(Lin et al., 2020)
Microbiota regulation	HFS-induced obesity	30 mg/kg	SCFAs	(Etxeberria et al., 2015)
	Intestinal barrier damage-induced disorder	0.4 mg/kg diet	SCFAs	(Feng et al., 2023)
	HFD-induced disorder	200 mg/kg	SCFAs	(Xie et al., 2023)

### 3.1 Alleviation of ulcerative colitis

Ulcerative colitis (UC) is a chronic, relapsing inflammatory bowel disease. It is characterized by persistent or recurrent mucosal inflammation primarily confined to the colon and rectum (Gros and Kaplan, 2023). Immune system dysregulation plays a critical role in UC. It is closely linked to both the pathogenesis and progression of the disease (Tang et al., 2025). Consequently, immunomodulation has emerged as a therapeutic strategy for UC (Parada Venegas et al., 2019). Preclinical studies have reported protective effects of QE in animal models of UC, owing to its anti-inflammatory, antioxidant, and immunomodulatory properties.

Regarding the mitigation of inflammation and oxidative stress, Wei et al. found that 50 mg/kg QE reduced the expression of pro-inflammatory cytokines, including IL-6 and interleukin-17A (IL-17A), in the colonic tissues of DSS-induced UC mice, while decreasing the levels of the neutrophil marker Ly6G and the antioxidant enzyme SOD2. They demonstrated that QE inhibited the activation of the NF-κB signaling pathway, thereby ameliorating UC (Wei et al., 2025). Jiang et al. reported that daily administration of 10 mg/kg QE to mice with 3% DSS-induced UC not only reduced colonic levels of TNF-α and IL-1β, but also suppressed NF-κB pathway activation, thereby alleviating colitis symptoms (Jiang Z. et al., 2024). However, neither study assessed whether these anti-inflammatory effects translate into sustained histological or barrier repair. In another study, Yin et al. demonstrated that 100 mg/kg QE ameliorated ochratoxin A-induced UC in rats by reducing colonic levels of MDA, NO, myeloperoxidase (MPO), and hydroxyproline, while elevating SOD and GSH levels. QE downregulated the expression of several pro-inflammatory and tissue injury-related genes, including osteopontin (OPN) and IL-6, thereby exerting protective effects against colonic injury. Whether the protective effects of QE observed in this mycotoxin-driven model can be extrapolated to human UC, which is primarily immune-mediated and chronic in nature, requires further investigation (Yin et al., 2023). In addition, Wang et al. reported that in mice with 3% DSS-induced UC, 25–100 mg/kg QE reduced UC by upregulating the expression of the tight junction proteins zonula occludens-1 (ZO-1) and Claudin-1 in colonic tissues. This intestinal barrier-protective effect was mediated by AhR activation. This resulted in three downstream consequences. They comprised suppression of pro-inflammatory signaling, attenuation of intestinal permeability, and diminution of bacterial translocation. However, the role of AhR was validated primarily through pharmacological antagonism, and no AhR gene knockout or knockdown models were employed, as acknowledged by the authors themselves (Wang et al., 2024). In a DSS-induced mouse model of UC, administration of 100 mg/kg QE was found to reduce colonic levels of TNF-α, IL-6, and IL-1β. This pharmacological effect was accompanied by the inhibition of the PI3K/Akt signaling pathway. Only a single dose of 100 mg/kg QE was tested in the *in vivo* experiments. Whether lower or higher doses produce comparable or greater anti-colitis effects, or whether a dose-dependent relationship exists, remains to be determined (Zhang et al., 2022). Gupta et al. reported that the hydroalcoholic extract of S. grandiflora, in which QE was identified and quantified as one of the major flavonoids, reduced the disease activity index (DAI) and colonic damage in acetic acid-induced UC models by suppressing TNF-α and IL-6 levels, highlighting its anti-inflammatory potential against UC. However, the protective effects were attributed to the extract as a whole. The specific contribution of QE within this multi-component extract was not individually assessed (Gupta et al., 2018). Gao et al. demonstrated that 30 mg/kg QE ameliorated DSS-induced UC in mice. This was achieved by suppressing the cyclic GMP-AMP synthase (cGAS)/stimulator of interferon genes (STING) pathway. This inhibition reduced colonic levels of the M1 macrophage marker iNOS and pro-inflammatory factors, including TNF-α, interferon-beta (IFN-β), and C-X-C motif chemokine ligand 10 (CXCL10). It promoted the expression of the M2 macrophage marker arginase-1 (Arg1) and anti-inflammatory cytokines such as interleukin-10 (IL-10) and C-C motif chemokine ligand 17 (CCL17). By restoring the M2/M1 macrophage homeostatic balance, QE alleviated DSS-induced colitis. While QE suppressed the cGAS-STING pathway in colonic tissue, the upstream source of dsDNA that activates this pathway *in vivo* was not examined. It therefore remains unclear whether QE acts by reducing dsDNA release or by inhibiting cGAS-STING signaling (Gao et al., 2024).

### 3.2 Alleviation of colorectal cancer

Colorectal cancer (CRC) ranks among the most prevalent malignancies worldwide. Its primary risk factors encompass family history, intestinal polyps, and UC (Fadlallah et al., 2024; Wan et al., 2020). In this context, accumulating evidence has reported that QE induces apoptosis in human CRC cells.

Yang et al. demonstrated that 50–200 μmol/L QE induced apoptosis and G0/G1 cell cycle arrest in HT-29 cells by inhibiting Akt phosphorylation. This intervention decreased c-Myc and COP9 signalosome subunit 6 (CSN6) expression, upregulated p53 and Bax, and downregulated Bcl-2. This study was conducted exclusively in HT-29 cells. Whether the Akt-CSN6-Myc axis mediates comparable anti-colorectal cancer effects in other colon cancer cell lines or *in vivo* models remains to be determined (Yang et al., 2016). Raja et al. reported that 40 μmol/L QE induced apoptosis in HT-29 cells. This effect was achieved by modulating cyclooxygenase-2 (COX-2) and iNOS expression, promoting NF-κB nuclear translocation, and enhancing ROS generation. This pro-apoptotic effect was attenuated upon COX-2 silencing. This indicated that the anticancer activity of QE was COX-2 dependent. QE exhibited no cytotoxicity toward normal intestinal epithelial cells, highlighting its favorable safety profile and potential for further investigation in CRC models. The COX-2 dependency of QE-induced apoptosis was supported primarily by COX-2 knockdown in HT-29 cells. COX-2 overexpression in COX-2-negative cells was not tested to see if it restored sensitivity to QE, so the proposed COX-2-dependent mechanism requires further validation (Raja et al., 2017). Another study revealed that 50 μmol/L QE suppressed the PI3K/Akt/mTOR survival pathway in Caco-2 and DLD-1 colon cancer cells. It also activated the JNK/JUN pro-apoptotic cascade in these cells. QE inhibited β-catenin nuclear accumulation and c-Myc expression. These effects led to suppressed proliferation and induced apoptosis. These findings were obtained exclusively in cultured colon cancer cells. No *in vivo* animal model was employed to confirm that QE suppresses colon cancer progression through these pathways (Refolo et al., 2015). Zhang et al. demonstrated the anti-CRC activity of 150 μmol/L QE in HCT-116 cells. The intervention promoted apoptosis and inhibited proliferation through alterations in non-coding RNA and mRNA expression profiles. These cellular responses were associated with the modulation of the PI3K/Akt and Ras signaling pathways, alongside the downregulation of key genes such as leucine-rich alpha-2-glycoprotein 1 (LRG1). This study relied primarily on transcriptomic profiling and bioinformatics analysis. No functional validation experiments were performed to confirm that the identified non-coding RNAs or LRG1 are causally involved in the anti-CRC effects of QE (Zhang et al., 2019). Lin et al. demonstrated the anti-tumorigenic effect of 30 mg/kg QE in AOM/DSS-induced CRC mouse models. QE supplementation suppressed colon tumorigenesis through attenuation of oxidative stress and inflammation. This was evidenced by modulated levels of redox and oxidative markers, including lipid peroxidation (LPO), NO, SOD, glucose-6-phosphate dehydrogenase (G6PD), and GSH. Furthermore, QE intervention decreased both tumor number and size while promoting the recovery of the hematopoietic system and inhibiting colon cancer development. Importantly, one point to consider is that the study design did not include a QE-only arm, so the possibility that QE exerts baseline modulatory effects on colonic redox signaling and tumor-related pathways independent of AOM/DSS challenge cannot be entirely excluded (Lin et al., 2020).

### 3.3 Regulation of the microbiota

In recent years, the gut microbiota has emerged as a central focus in the pathogenesis of various systemic and local diseases (Adak and Khan, 2019). As a complex microbial ecosystem colonizing the gastrointestinal tract, the gut microbiota orchestrates host physiological homeostasis and overall health. Beyond participating in nutrient metabolism and energy homeostasis, it maintains intestinal barrier integrity and modulates immune functions (Chen et al., 2021). Accumulating evidence indicates that gut microbiota dysbiosis is linked to the onset and progression of diverse intestinal disorders.

As a naturally occurring flavonoid, QE has been reported to show protective effects by maintaining gut microbiota homeostasis. An HFD disrupts this microbial balance, leading to intestinal dysbiosis and the subsequent development of metabolic disorders (Yang et al., 2022). Etxeberria et al. reported that oral administration of 30 mg/kg/day QE to rats with high-fat sucrose diet (HFS)-induced obesity increased the abundance of short-chain fatty acid (SCFA)-producing bacteria, promoting intestinal metabolism. However, SCFA measurements were conducted *ex vivo* in feces, which may not accurately reflect the real production of SCFAs (Etxeberria et al., 2015). LPS stimulation compromises the intestinal barrier. Feng et al. investigated the pharmacological effects of dietary QE at 0.4 mg/kg in an LPS-induced intestinal inflammation model in laying hens. Their findings demonstrated that QE enriched SCFA-producing bacteria, reshaping the gut microbiota ecosystem. Furthermore, QE upregulated the tight junction proteins Claudin-1 and Occludin, alongside the mucin protein Mucin2, ultimately alleviating LPS-induced barrier damage. The observed enrichment of SCFA-producing bacteria was not accompanied by direct measurements of cecal or fecal SCFA concentrations. The proposed link between microbial shifts and barrier protection therefore requires further support (Feng et al., 2023). Similarly, 200 mg/kg QE ameliorated metabolic syndrome in HFD-fed mice, an effect closely linked to enhanced bacterial production of SCFAs (such as butyrate) and improved intestinal energy metabolism. However, this study did not validate the causal role of specific bacteria in driving SCFA production or intestinal energy metabolism, as no germ-free, single-strain colonization or FMT experiments were performed, leaving the underlying mechanisms to be confirmed (Xie et al., 2023). Expanding on these mechanisms, Xiong et al. demonstrated that 50–100 mg/kg QE alleviated DSS-induced UC in mice by modulating gut microbiota composition and restoring tryptophan metabolism. Notably, QE increased indole-3-lactic acid levels to activate the AhR pathway and reinforce intestinal barrier integrity. Although the authors confirmed that QE’s protective effects were gut microbiota-dependent through FMT, they did not causally validate which specific bacterial taxa are responsible. This gap highlights the need for further studies using germ-free mice or single-strain colonization to pinpoint the microbial mediators and to further validate the QE–microbiota–metabolite–AhR axis in a causal setting (Xiong et al., 2025). Consistently, Lei et al. demonstrated that 100 mg/kg QE enriched beneficial Clostridium XIVa and XI in colitis mice, driving the microbial production of isovanillic acid (IVA) to restore barrier integrity and suppress inflammation. Moreover, antibiotic depletion experiments confirmed that the anti-colitis effects of QE were largely microbiota-dependent. The loss of gut microbes abolished both IVA generation and protection, underscoring the essential role of microbial metabolism in the pharmacological activity of QE. Although antibiotic depletion established the necessity of gut microbiota, the specific bacterial species responsible for converting QE into IVA were not identified, as acknowledged by the authors themselves in this study. Future studies isolating and culturing candidate Clostridium strains could help clarify which bacteria are responsible for this conversion (Lei et al., 2025).

## 4 Integrative mechanisms underlying the pleiotropic effects of QE

Considered collectively across different hepatic and intestinal disease models, the signaling pathways regulated by QE exhibit considerable disease- and tissue-specificity while remaining mechanistically interconnected. These pathways converge on several core biological processes: redox homeostasis, inflammatory sensing, mitochondrial function, cell death, and tissue remodeling. The following integrative analysis aims to synthesize these convergent patterns into a coherent mechanistic framework. However, this framework represents an inductive synthesis of available correlative evidence from preceding sections rather than an established causal paradigm, and its validity awaits confirmation through orthogonal and rigorous investigations.

Oxidative stress represents a major convergence node of QE activity. Across multiple hepatic injury models, QE decreases ROS and MDA levels while restoring antioxidant defenses like GSH, SOD, and CAT (Huang et al., 2023; Kabirifar et al., 2017). In several models, these antioxidant effects are accompanied by the modulation of Nrf2/ARE, HO-1, and NOX-related signaling (Fawzy et al., 2023; Truong et al., 2016). These findings indicate that QE modulates the cellular redox environment, thereby influencing downstream stress-responsive and inflammatory pathways. Because redox imbalance is coupled to inflammatory signaling, pathways like TLR4/NF-κB, MAPK, and NLRP3 sense or amplify cellular stress driven by oxidative imbalance, damage-associated signals, and microbial products. In cholestatic liver injury, QE targets the Rac1/NOX1, NF-κB, and TGF-β/Smad axes (Kabirifar et al., 2017; Lin et al., 2014). In other hepatic injury models, coordinated changes in Nrf2, NLRP3, and apoptosis-related proteins emerge (Atta et al., 2025). These observations indicate that oxidative stress, inflammation, and cell death form mutually reinforcing pathological circuits, and that multi-target intervention within these circuits yields broader tissue protection.

Similar cross-talk characterizes fibrotic and tumor-associated signaling, where TGF-β/Smad acts as a major driver of hepatic stellate cell activation and extracellular matrix deposition. NF-κB, MAPK, and PI3K/Akt signaling also participate in inflammatory responses, cell survival, and fibrogenesis. In hepatic fibrosis models, QE inhibits both TGF-β1/Smad2/3 and PI3K/Akt (Wu et al., 2017b). In HCC models, QE inhibits JAK2/STAT3 and alters the expression of Bcl-2, Bax, Beclin-1, and other apoptosis- or autophagy-related proteins (Ji et al., 2019; Wu et al., 2019). These findings are consistent with coordinated regulation of inflammatory signaling, cell survival, and cell-death programs. QE contains multiple phenolic hydroxyl groups, a structural feature associated with its redox-modulating properties (Carrillo-Martinez et al., 2024). However, the pharmacological actions of QE extend far beyond ROS scavenging, encompassing the modulation of kinase signaling, transcriptional regulation, mitochondrial function, inflammasome activity, and cellular metabolism. The relative contribution of each mechanism depends on tissue context, disease stage, cell type, dose, and the local exposure of QE and its metabolites.

## 5 Connection of QE to gut-liver system diseases

### 5.1 Physiological connection between the liver and intestine

Anatomically, the portal vein and biliary system closely link the liver and intestine. The portal circulation transports digestive nutrients, alongside gut-derived microbial products and toxins, to the liver. Serving as a physiological “firewall,” the liver efficiently clears translocation-prone commensal microorganisms and their toxins before delivering metabolized nutrients into the systemic circulation (Albillos et al., 2020). A complex circuitry of signaling pathways drives the bidirectional communication within the gut-liver axis. For instance, the gut microbiota regulates bile acid metabolism. These acids function as signaling molecules to modulate hepatic lipid metabolism and restrict aberrant lipid accumulation (Roberts et al., 2002). Consequently, these two organs exhibit profound functional interdependence. As the first checkpoint for portal-derived bacteria and metabolites, the liver provides a crucial immunological barrier against systemic infection and serves as the primary biotransformation site (Kubes and Jenne, 2018). Mechanistically, hepatocytes influence the composition and function of the gut microbiota through the synthesis and secretion of bile acids and other metabolites, thus preserving gut microbial homeostasis (Nicholson et al., 2012; Winston and Theriot, 2020). The gut microbiota plays a key role in the enterohepatic circulation of bile acids. It biotransforms primary bile acids, which are synthesized by hepatocytes, into secondary bile acids. Approximately 95% of these bile acids are reabsorbed in the distal ileum and transported back to the liver via the portal circulation, thereby completing the enterohepatic cycle (Marrero et al., 2018). The liver and gut function as an integrated, interdependent unit through the gut-liver axis rather than as isolated organs. This functional unit is essential for the maintenance of systemic physiological homeostasis.

### 5.2 Pathological connection between the liver and intestine

Clinical studies consistently link immune-mediated liver diseases with concomitant UC, suggesting shared immunoregulatory mechanisms in their pathogenesis (Wang et al., 2021). The human gastrointestinal tract harbors a vast microbial community essential for metabolic homeostasis, immune surveillance, and intestinal barrier integrity (Neurath et al., 2025). Gut microbiota dysbiosis instigates various hepatic disorders primarily through oxidative stress, inflammatory cascades, and aberrant lipid metabolism (Cao, 2018). Therapeutic modulation of the gut microbiota has become a promising approach for mitigating experimental hepatic injury. Chronic liver diseases cover a pathological spectrum that ranges from steatosis and hepatitis to progressive fibrosis, cirrhosis, and HCC. These diseases are triggered by diverse etiologies, including hazardous alcohol consumption, xenobiotics, viral infections, hepatotoxins, and obesity. Chronic alcohol abuse damages hepatocytes. It also disrupts intestinal barrier integrity via the induction of enterocyte apoptosis and the degradation of tight junction proteins. This compromised barrier facilitates portal translocation of endotoxins, thereby amplifying hepatic sterile inflammation and accelerating disease progression (Milosevic et al., 2019). Distinct dysbiotic profiles are recognized as primary drivers of MASLD, acting independently of or synergistically with metabolic risk factors such as obesity. Taxonomic variations at the phylum, genus, and species levels drive interindividual differences in host metabolic responses to high-fat diets, thereby modulating MASLD susceptibility (Chen and Vitetta, 2020). Under persistent injury, unresolved liver inflammation transitions into fibrogenesis and eventual cirrhosis. This progressive architectural remodeling, driven by the excessive deposition of extracellular matrix metabolites like fibrillar collagens, impairs parenchymal function and primes the microenvironment for HCC initiation (Sorribas et al., 2019). Pathologically, gut-liver axis disruption serves as a central catalyst for fibrogenesis. In BDL-induced hepatic fibrosis models, the translocation of viable gut microbes or their metabolites to the liver activates tonic type I interferon (IFN) signaling in hepatic myeloid cells. Intracellular bacterial recognition prompts these myeloid cells to secrete excessive type I IFNs, which engage their cognate receptors to drive immunosuppressive IL-10 expression. This altered hepatic immune microenvironment compromises the clearance of gut-derived pathogens. This immune cascade establishes a self-perpetuating vicious cycle that accelerates cirrhosis and its systemic complications (Hackstein et al., 2017).

### 5.3 Effects of QE on the gut-liver axis

#### 5.3.1 Bile acids

Hepatocytes synthesize bile acids from cholesterol and secrete them into the biliary system. Upon postprandial stimulation, these bile acids enter the intestinal lumen via bile flow. The majority are reabsorbed in the terminal ileum and transported back to the liver through the portal vein. This completes the enterohepatic circulation (Perino and Schoonjans, 2022). Within the intestinal tract, bile acids not only facilitate the emulsification and absorption of dietary lipids but also exert intrinsic antimicrobial activities. These actions shape the gut microbiota composition by modulating the luminal microenvironment. The gut microbiota biotransforms these primary molecules into diverse secondary bile acids, reducing their cytotoxicity and maintaining metabolic homeostasis (Begley et al., 2005). Accumulating evidence indicates that QE is involved in bile acid metabolism, suggesting its pharmacological effects in enterohepatic diseases. QE alleviates cholestasis by inhibiting excessive bile acid accumulation, thereby exerting hepatoprotective effects. Fawzy et al. reported that QE reduced serum transaminase and bilirubin levels, thereby ameliorating cholestatic liver injury (Fawzy et al., 2023). Hepatic innate immune cells sense gut-derived signals via bile acid-activated receptors such as FXR and TGR5, thereby orchestrating immune defense against microbial invasion. This mechanistic link suggests that QE may alleviate gut-liver axis-associated pathologies by modulating these bile acid-dependent immunological cascades (Lin et al., 2014).

#### 5.3.2 FXR/TGR5

In addition to facilitating dietary lipid digestion and absorption, bile acids act as critical signaling moieties that govern homeostasis along the gut-liver axis. Within this network, FXR and TGR5 are the main mediators of bile acid signaling (Jia et al., 2018). These receptors are expressed in both the liver and gut. They preserve barrier integrity and modulate bile acid pool sizes, energy metabolism, and inflammatory responses (Baars et al., 2015; You et al., 2025). Within human hepatocytes, metabolic homeostasis is governed by the FXRα2 isoform. Its transcriptional activity depends on the ER-2 motif within specific DNA response elements (Van Der Veen et al., 2025). Emerging evidence highlights that the hepatoprotective and gut-barrier-preserving properties of QE are coupled with its modulation of FXR and TGR5 signaling cascades. By targeting these receptors, QE suppresses key pro-inflammatory and pro-fibrotic mediators, including TNF-α, NF-κB, and TGF-β1. This mitigates tissue injury and restores homeostatic balance. Under pathological conditions such as viral hepatitis and enteritis, QE intervention blunts pro-inflammatory cytokine release, halts NF-κB activation, and curtails hepatocyte apoptosis and necroptosis. This is associated with tissue-protective effects. FXR activation exerts potent anti-inflammatory actions and sustains mucosal and hepatic immune tolerance (Du et al., 2025). The pharmacological effects of QE against hepatic injury and cholestasis are inherently linked to its pharmacological activation of the FXR pathway. FXR activation may alleviate cholestatic injury by stimulating hepatocyte autophagy, which facilitates the clearance of toxic intracellular bile acids. Through this FXR-dependent axis, QE may restrict the progression of liver fibrosis and CRC. FXR regulates the expression of gene networks by governing bile acid, lipid, glucose, and amino acid metabolism (Massafra et al., 2018). This may contribute to the reported effects of QE on hepatic steatosis and lipid accumulation in MASLD models.

#### 5.3.3 The microbiota

The gut microbiota is recognized as a central pathological factor. It plays a key role in the initiation and progression of various enterohepatic diseases. QE ameliorates these disorders by stimulating the production of SCFAs, remodeling the taxonomic composition and functional capacity of the gut microbiota, and restoring microbial homeostasis (Feng et al., 2023; Maslin et al., 2022). QE-mediated regulation of the bile acid pool exerts feedback effects on the gut microbiota. This reflects the bidirectional nature of the gut-liver axis. Intestinal microbes biotransform host-derived primary bile acids into secondary bile acids. These secondary bile acids are actively reabsorbed in the intestine and transported back to the liver to sustain the enterohepatic circulation. By regulating this bile acid metabolism and downstream gut-liver signaling, the microbiota maintains homeostatic equilibrium. QE harnesses this symbiotic network to exert hepatoprotective and intestinal-barrier-preserving effects by modulating bile acid profiles and microbial composition (Chopyk and Grakoui, 2020).

QE confers protection against enterohepatic pathologies by remodeling the gut microbiota and modulating bile acid metabolism. These actions subsequently orchestrate FXR- and TGR5-dependent signaling cascades across the gut-liver axis.

However, it remains to be verified whether the protective effects of QE are mediated via the gut-liver axis and whether the gut microbiota acts as a causal driver or merely a bystander in this process. Porras et al. first revealed the liver-protective effect of QE through the gut-liver axis in a mouse model of MASLD induced by an HFD. The study found that the dysbiosis of the gut microbiota induced by HFD led to damage of the intestinal barrier, elevated plasma LPS and endogenous ethanol levels, which activated the liver TLR4-NF-κB signaling pathway and NLRP3 inflammasome, aggravating fatty liver. QE intervention could reverse the dysbiosis, restore the production of SCFAs, repair the intestinal barrier, and reduce endotoxemia, thereby blocking the liver inflammation pathway and ultimately improving liver fat accumulation and damage. The causal role of gut microbiota in mediating the protective effects of QE was not tested, as no microbiota transplantation, antibiotic depletion, or germ-free mouse experiments were performed. Whether the gut microbiota acts as a causal driver or merely a bystander in these processes remains to be verified (Porras et al., 2017).

Based on this, Porras et al. further established the causal role of the gut microbiota through a fecal microbiota transplantation experiment. Germ-free mice colonized with microbiota from HFD responders exhibited pronounced liver injury and inflammatory activation. Conversely, recipients of microbiota from HFD non-responders or QE-treated donors demonstrated reduced endotoxemia following HFD challenge, alongside suppressed hepatic TLR4 and NLRP3 expression and near-normal histology. This indicated that a protective microbiota effectively halted HFD-induced aberrant activation of the gut-liver axis. This study further found that the specific bacterial composition induced by QE was the key medium for its protective effect through the gut-liver axis. QE exerted its liver-protective and anti-inflammatory benefits through reshaping the composition of the gut microbiota (especially enriching Akkermansia and inhibiting Helicobacter), restoring the function of the intestinal barrier, reducing the translocation of endotoxin through the portal vein to the liver, and thereby blocking the excessive activation of the TLR4-NLRP3 inflammatory pathway, ultimately exerting its protective effects via the gut-liver axis. However, while this study identified Akkermansia as a genus strongly correlated with the protective phenotype, no single-strain colonization or depletion experiment was performed to confirm that Akkermansia was causally required for these protective effects (Porras et al., 2019).

## 6 Discussion and outlook

QE is a naturally occurring flavonoid. It is widely distributed among diverse botanical sources. It has a variety of biological properties, including anti-inflammatory, antioxidant, and antitumor activities, thereby showing pharmacological effects in preclinical models of enterohepatic pathologies. By evaluating the pharmacological effects of QE within the enterohepatic system, this review establishes a theoretical foundation for subsequent experimental research and clinical translation. Current literature reveals that QE ameliorates enterohepatic disorders through intricate multi-target and multi-pathway mechanisms. It regulates critical physiological responses, encompassing oxidative stress, inflammatory cascades, immune homeostasis, cellular apoptosis, and metabolic pathways.

Current evidence indicates that QE has broad-spectrum hepatoprotective effects against various liver disorders. Reported *in vivo* dosages range from 10 to 200 mg/kg, while corresponding *in vitro* doses range from 5 to 400 μmol/L. At these pharmacological levels, QE mitigates hepatic injury, alleviates cholestasis and fibrotic remodeling, and impedes the pathogenesis of MASLD, MASH, and HCC. Mechanistic investigations reveal that QE activates the Nrf2/ARE signaling axis to increase antioxidant enzymes such as SOD. This activation attenuates oxidative stress and inhibits the secretion of pro-inflammatory cytokines like IL-6 and IL-1β. Concurrently, it regulates apoptosis-related proteins, particularly Bax, caspase-3, and p53, to halt programmed cell death and confer hepatic protection. The resolution of cholestasis is linked to the modulation of the Nrf2 and NF-κB pathways, alongside the regulation of Rac1, SOD, and CAT. QE restricts tissue remodeling in liver fibrosis driven by cytokines like TNF-α, IL-1β, and IL-6. This is achieved by quenching oxidative stress and blocking hepatic stellate cell activation. For metabolic disorders like MASLD and MASH, QE exerts modulatory effects by regulating mitochondrial biogenesis genes and inhibiting TLR4 signaling, diminishing these aforementioned pro-inflammatory mediators. Its pharmacological effects against viral hepatitis rely on complex networks encompassing the PI3K/Akt and PI3K/Akt/GSK3β cascades, the METTL3-dependent PTEN mRNA m^6^A methylation axis, and mitochondrial apoptotic pathways. In addition to its direct cytotoxicity, QE has been reported to reduce HCC progression in preclinical models by regulating JAK2/STAT3 signaling, downregulating Bcl-2, and upregulating Beclin-1 to promote apoptosis and autophagy in HCC cells (Figure 1).

**FIGURE 1:**
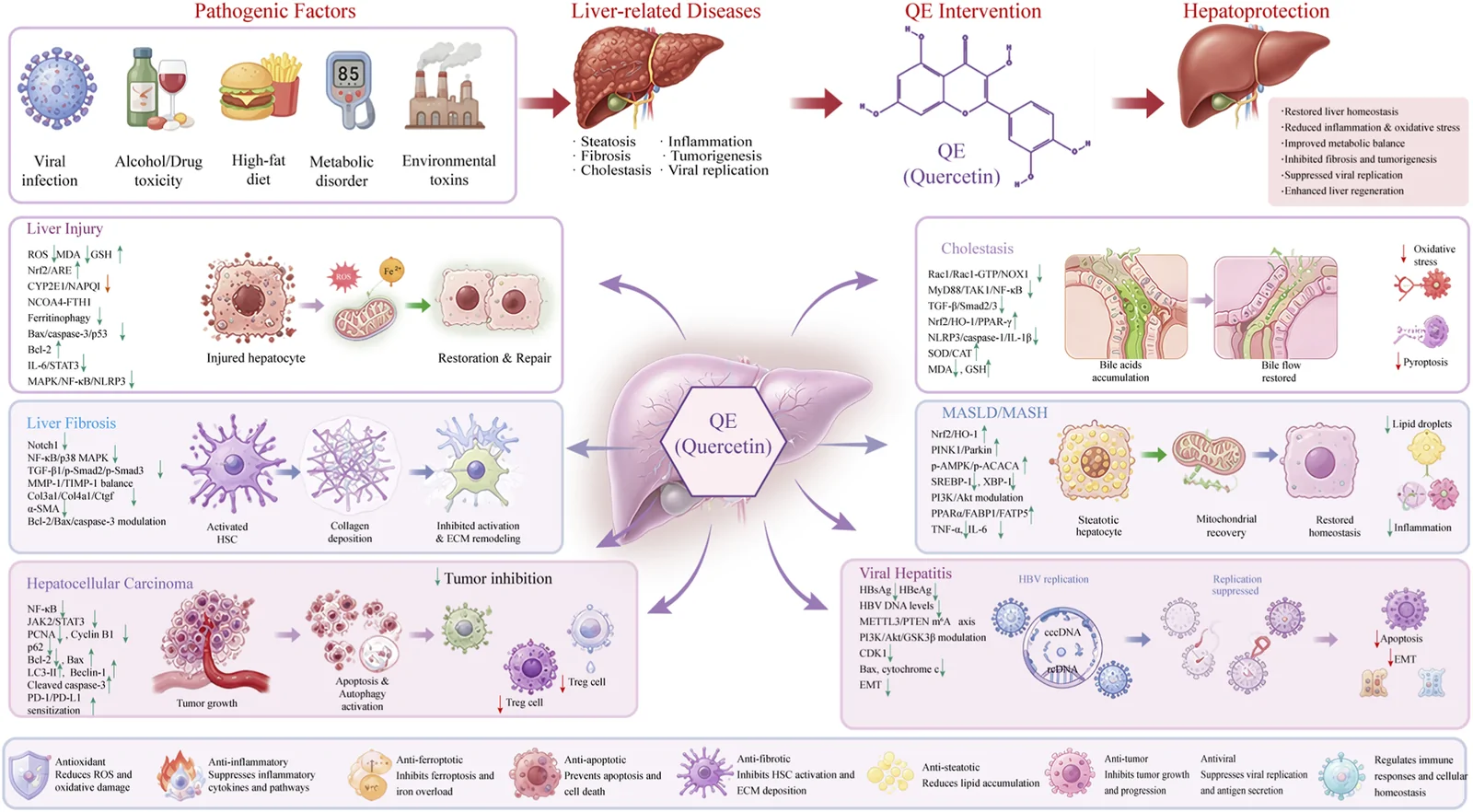
The pharmacological effects of QE in liver disease models.

Relevant models show that QE has pharmacological effects in alleviating UC, inhibiting CRC progression, and regulating the microbiota at *in vivo* doses of 10–100 mg/kg and *in vitro* concentrations of 50–200 μmol/L. These reported effects are primarily driven by the suppression of inflammation and oxidative stress. They also involve the modulation of host immune responses. QE alleviates UC by downregulating pro-inflammatory cytokines such as IL-1β, TNF-α, and IL-6, while inhibiting the cGAS-STING and PI3K/Akt signaling pathways. In CRC, QE inhibits tumor progression by inducing G1-phase cell cycle arrest and altering the expression profiles of multiple non-coding RNAs and mRNAs. These antitumor properties are mechanistically linked to the Akt-CSN6-Myc signaling axis and the JNK/JUN pathway. In addition, QE promotes the production of SCFAs, which restore gut microbial homeostasis, improve intestinal barrier integrity, and confer protection against intestinal pathologies (Figure 2).

**FIGURE 2:**
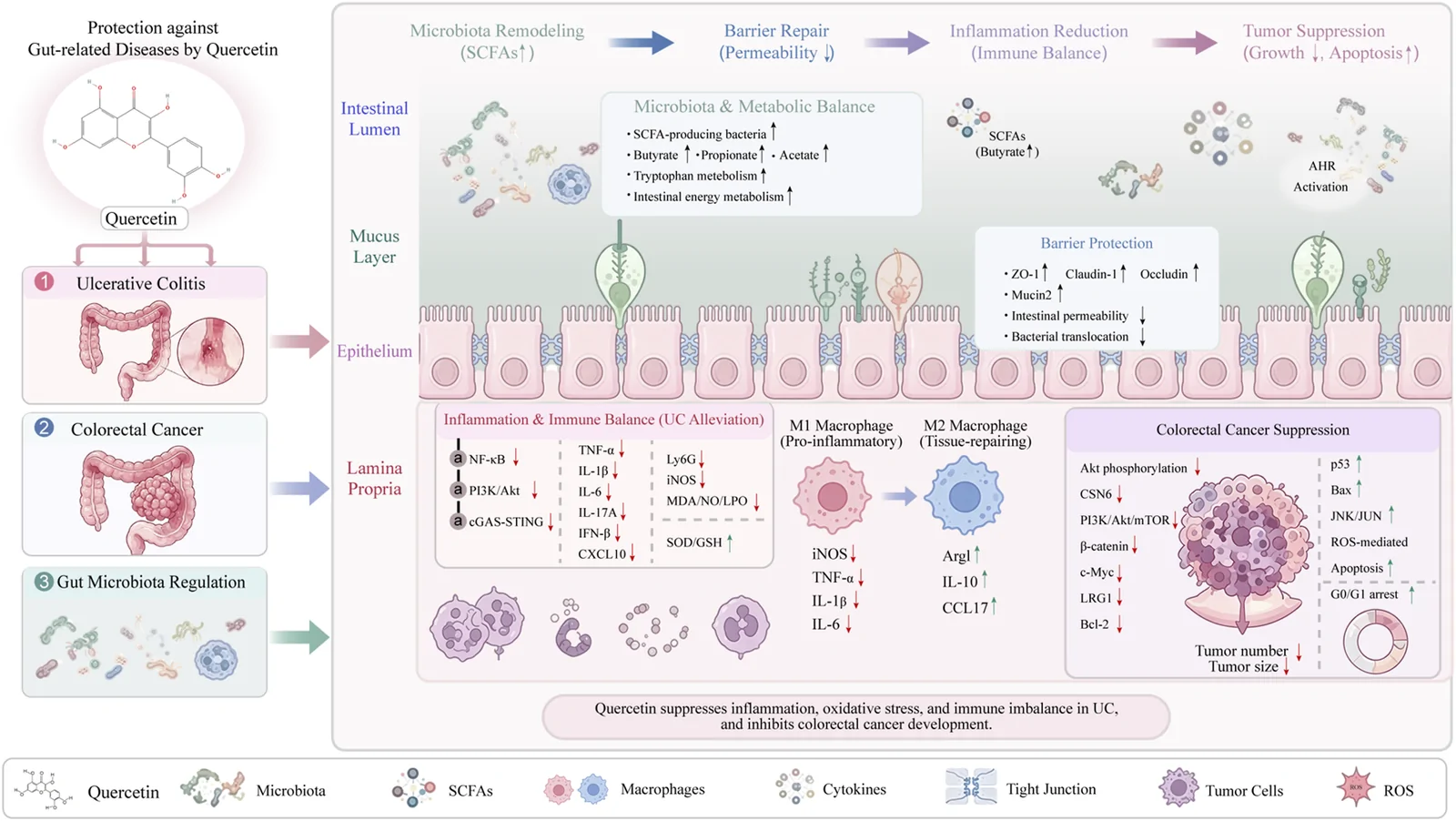
The pharmacological effects of QE in gut disease models.

Integrating the hepatic and intestinal actions delineated above (Figures 1, 2), the pleiotropic pharmacological effects of QE can be unified through the lens of the gut-liver axis. As schematically illustrated in Figure 3, QE orchestrates a multi-tiered regulatory network: it reshapes the gut microbiota (e.g., enriching Akkermansia and SCFA producers), restores intestinal barrier integrity, curtails endotoxin translocation, and modulates bile acid profiles, which collectively engage FXR/TGR5 signaling to quell hepatic inflammation, steatosis, and fibrogenesis. This framework highlights the gut-liver axis as a key mediator of QE’s pharmacological activities in preclinical enterohepatic models.

**FIGURE 3:**
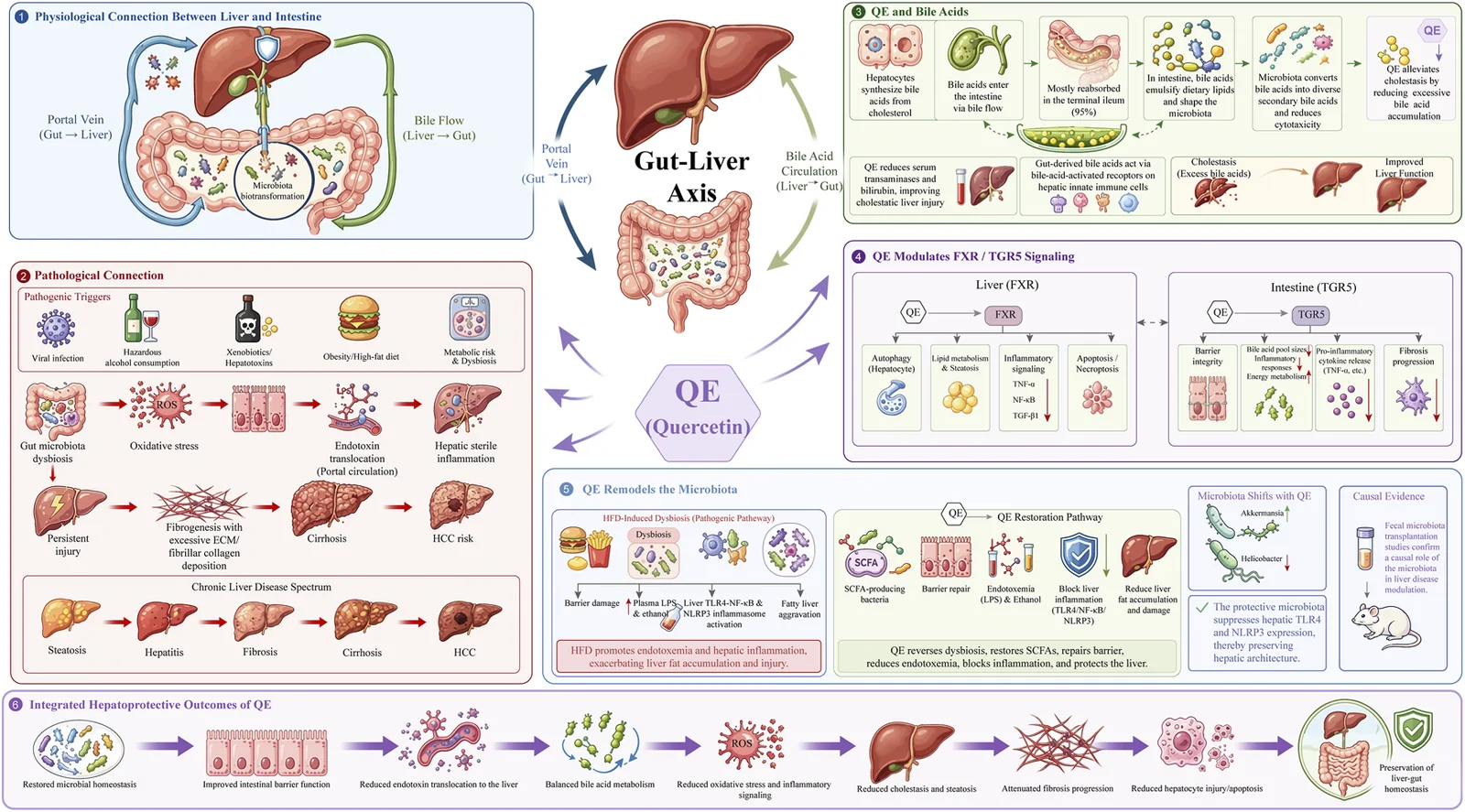
The gut-liver axis as a therapeutic hub for QE.

QE confers protection against enterohepatic diseases by modulating gut microbiota-associated bile acid metabolism and FXR/TGR5-mediated gut-liver axis signaling. QE upregulates the activity of bile acid-metabolizing enzymes and important receptors like FXR and TGR5 to reduce cholestasis. QE stimulates the production of SCFAs, reshapes the taxonomic composition and functional capacity of the gut microbiota, and restores microbial homeostasis, ultimately ameliorating gut-liver axis-associated pathologies.

Despite the low oral bioavailability of QE, its pharmacological effects are observed *in vivo*. This apparent paradox is reconciled by first-pass metabolism. It is worth noting that the effective QE concentrations used in most *in vitro* cell experiments are much higher than the actual metabolite levels that can be reached in plasma after oral administration. Therefore, interpreting *in vitro* results demands careful consideration of actual *in vivo* exposure levels. Following ingestion, QE and its metabolites accumulate in the intestinal wall and liver during first-pass metabolism, resulting in local concentrations that are higher than corresponding plasma levels (Chen et al., 2005). Thus, the higher concentrations used in *in vitro* experiments to some extent simulate the actual exposure conditions in the target organs within the absorption window. Meanwhile, unabsorbed QE and its glycosides are continuously biotransformed by the gut microbiota into various active phenolic acids, such as 3,4-dihydroxybenzeneacetic acid, protocatechuic acid, and isorhamnetin. These metabolites reach exceptionally high local concentrations in the large intestine. They repeatedly expose the liver via enterohepatic circulation, thereby sustaining pharmacological effects in enterohepatic tissues despite low systemic plasma levels (D’archivio et al., 2010; Zhu et al., 2024). The biological effects of QE and its metabolites in liver and intestinal tissues have been verified in multiple animal models: QE can reduce lipid accumulation in the liver and the expression of inflammatory factors, and regulate the structure of the gut microbiota such as reducing the Firmicutes/Bacteroidetes ratio and increasing the abundance of Akkermansia (Tan et al., 2021; Zhao et al., 2017). Furthermore, it improves liver insulin sensitivity by activating SIRT1 and inhibiting the NF-κB pathway (Dao et al., 2026). These overall effects are consistent with the molecular mechanisms revealed in cell models such as AMPK, Akt, and PPAR-γ. Based on the above analysis, the relatively higher concentrations used in in vitro experiments are necessary for experiments that amplify signals at the mechanism level, and the molecular pathways they reveal have clear physiological relevance under the local high exposure conditions in the liver and intestinal target organs; the overall effects in the body are the comprehensive manifestation of these mechanisms participating in the enrichment, metabolic transformation, and long-term accumulation at the local level, and the differences between the two precisely reflect the necessity and complementary value of research from molecular mechanisms to organ levels.

Extensive preclinical evidence shows that QE exerts protective effects through multi-target signaling networks, with suppression of inflammation and oxidative stress serving as its principal mode of action (Ma et al., 2020). However, certain limitations should be acknowledged. The studies included in this review cover multiple evidence levels ranging from *in vitro* cell experiments, animal models to a small number of human trials, and different levels of research need to be viewed differently when translating to clinical applications. It should also be noted that the inherent redox activity of polyphenolic flavonoids such as QE is known to cause interference in lipase-based assays, fluorescent probes such as DCFH-DA, and *in vitro* enzyme activity systems. As QE is frequently recognized as one of the pan-assay interference compounds (PAINS), this can lead to promiscuous false positives and an overestimation of its lipid-lowering and antioxidant effects (Bolz et al., 2021). Therefore, when interpreting the existing literature, one must be cautious about such methodological factors. On the other hand, clinical trials and combination therapy evaluations remain limited; existing clinical evidence is constrained by small sample sizes, single-center designs, and a lack of rigorously designed randomized controlled trials (RCTs). Furthermore, the proposed mechanisms have yet to be fully validated in patients (Liu et al., 2025). A further limitation concerns the heterogeneity of the preclinical evidence base itself. Most mechanistic claims summarized in Sections 2 and 3 rely on a limited number of cell lines or single rodent models; observed changes should therefore be regarded as correlative rather than causally validated, and network-pharmacology or molecular-docking predictions require orthogonal experimental confirmation before being read as evidence of species-level activity. Reporting of dose range, minimal effective concentration, intervention duration, and positive/negative controls is also uneven across cited studies, constraining direct cross-comparison. The mechanistic framework presented here should be regarded as an integrative working model whose nodes vary in evidence strength. In response to these limitations, future research should establish standardized “interference exclusion” experimental protocols, prioritize the use of the *in vivo* metabolites of QE (rather than the prototypical glycoside) for *in vitro* mechanism validation, and through causal verification experiments such as microbiota transplantation and antibiotic removal, clarify the driving role of the gut microbiota in the pharmacological effects of QE, while replicating existing findings in chronic, progressive, and more clinically relevant disease models, and ultimately provide high-level evidence for the clinical translation of QE through rigorous RCT studies designed with histological or gut-liver axis function biomarkers as endpoints. In the realm of drug development, novel derivatives with enhanced pharmacological profiles can be generated from the QE scaffold through structural modification using natural product chemistry, opening new avenues for drug discovery. Rapid advancements in multi-omics technologies, including genomics, transcriptomics, proteomics, and metabolomics, will unravel the complex regulatory networks underlying QE therapy, thereby providing a pharmacological basis for further evaluating QE in experimental enterohepatic models (Jiang et al., 2020).

Collectively, QE exhibits pleiotropic pharmacological activities across enterohepatic pathologies. This is achieved via the coordinated modulation of multiple signaling pathways and molecular targets, reflecting an intricate regulatory network. These multi-target interventions confer protection upon the gut-liver axis, underscoring the need for further preclinical and clinical validation. Despite these promising findings, research identifying the shared mechanisms and common targets driving its dual pharmacological effects in hepatic and intestinal tissues remains limited. Future investigations are thus warranted to unravel these cross-organ mechanisms and ultimately accelerate the clinical translation of QE.
